# Single-Conidium Encapsulation in Oil-in-Water Pickering Emulsions at High Encapsulation Yield

**DOI:** 10.3389/fchem.2021.726874

**Published:** 2021-11-29

**Authors:** Liliya Kotliarevski, Karthik Ananth Mani, Reut Amar Feldbaum, Noga Yaakov, Eduard Belausov, Einat Zelinger, Dana Ment, Guy Mechrez

**Affiliations:** ^1^ Department of Food Sciences, Institute of Postharvest and Food Sciences, Agricultural Research Organization (ARO), Volcani Institute, Rishon Lezion, Israel; ^2^ Institute of Biochemistry, Food Science and Nutrition, The Robert H. Smith Faculty of Agriculture, Food and Environment, The Hebrew University of Jerusalem, Rehovot, Israel; ^3^ Department of Ornamental Plants and Agricultural Biotechnology, Institute of Plant Science, Agricultural Research Organization (ARO), Volcani Institute, Rishon Lezion, Israel; ^4^ Department of Plant Pathology and Weed Research, Institute of Plant Protection, Agricultural Research Organization (ARO), Volcani Institute, Rishon Lezion, Israel

**Keywords:** titania, mineral oil, Pickering emulsion, fungi, conidia, single-cell encapsulation

## Abstract

This study presents an individual encapsulation of fungal conidia in an oil-in-water Pickering emulsion at a single-conidium encapsulation yield of 44%. The single-conidium encapsulation yield was characterized by analysis of confocal microscopy micrographs. Mineral oil-in-water emulsions stabilized by amine-functionalized titania dioxide (TiO_2_-NH_2_ or titania-NH_2_) particles were prepared. The structure and the stability of the emulsions were investigated at different compositions by confocal microscopy and a LUMiSizer^®^ respectively. The most stable emulsions with a droplet size suitable for single-conidium encapsulation were further studied for their individual encapsulation capabilities. The yields of individual encapsulation in the emulsions; i.e., the number of conidia that were individually encapsulated out of the total number of conidia, were characterized by confocal microscopy assay. This rapid, easy to use approach to single-conidium encapsulation, which generates a significantly high yield with eco-friendly titania-based emulsions, only requires commonly used emulsification and agitation methods.

## Introduction

Encapsulation of living cells at the individual level has recently attracted much attention. Single-cell encapsulation of cells has a wide range of applications including the quantitative determination of pathogens (An et al., 2020), the cultivation of uncultured microbes (Ishii et al., 2010), genomic analysis (Lan et al., 2017), (Zhang et al., 2017), high-throughput single-cell analysis (Liu et al., 2020), detection of heteroresistance in microorganisms (Lyu et al., 2018), protection formulation for biopesticides (Yaakov et al., 2018), drug delivery (He et al., 2005), (Orive et al., 2015), (Köster et al., 2008), (Yang et al., 2016), (Tikekar et al., 2013), vaccine development (Eyer, 2020), single-cell protein analysis (Yin and Marshall, 2012), whole-cell biocatalysts (de Carvalho, 2017) and whole-cell biosensor cell therapy (Youn et al., 2020), (Liu et al., 2019).

Single-cell isolation techniques separate individual cells physically from each other and/or from the matrix (e.g., soil particles) (Ishii et al., 2010), typically to protect the cells from external stress (Youn et al., 2020), (Park et al., 2015). Several methods of individual encapsulation of cells have been developed, including solvent evaporation (Lorenceau et al., 2005), interfacial polymerization (Arshady, 1989), layer-by-layer deposition (Oliveira et al., 2016), gel microdroplets (Ryan et al., 1995), drop based microfluidics (Chen and Lin, 2019), (Liu et al., 2020), Rahman-activated gravity (Youn et al., 2020), the introduction of catalytic templates onto cell surfaces (Park et al., 2015), manual dilution (Blainey, 2013), microfluidics (Blainey, 2013), micropipette (Blainey, 2013), microdroplet (Blainey, 2013), optical and optoelectronic tweezers (Blainey, 2013), flow cytometry (Blainey, 2013), and Pickering emulsion technology (Yaakov et al., 2018).

Two immiscible liquids can be combined through a process of emulsification by mixing surfactants; i.e., amphiphilic molecules that self-assemble at the liquid-liquid interface and lower the surface tension of the emulsion droplets, or by mixing solid colloidal particles that adsorb droplets at the surface of the emulsion by partially wetting the solid by the two liquids. The former is known as classical or conventional emulsion, whereas the latter is labelled a Pickering emulsion (Yaakov et al., 2018), (Li et al., 2017), (van Wijk et al., 2014), (Wei et al., 2016), (Sharkawy et al., 2020). Droplet diameter is a function of particle size and composition (oil/water ratio and NPs content) (Zhang et al., 2016), (Zhang and Wang, 2016), (Anupam, 2017), (French et al., 2015), (French et al., 2016). Pickering emulsions were extensively studied in the 20th century. Their high colloidal stability and controllable droplet size make them particularly suitable for single-cell encapsulation (Yaakov et al., 2018), (Bashir et al., 2016), (Chen et al., 2015), (Röllig et al., 2019). The stability of Pickering emulsions is governed by different parameters including their particle concentration, the wettability of the particles (oil/water volume fraction), and destabilization mechanisms (Ortiz et al., 2020). The contact angle θ, at which the liquid-vapor interface meets the solid-liquid interface defines the degree of wettability. Note however, that the adsorption of solid particles at the interface is also dependent on particle size. The change in free energy (ΔG) associated with the desorption of a spherical particle from the oil/water interface to either of these bulk phases can be formulated as (Ortiz et al., 2020):

$$\Delta G=\pi r^{2}\prime Yow{\left(1\pm \cos\theta \right)}^{2}$$

where r stands for the particle radius, and Ύ_ow_ corresponds to the bare oil-water interfacial tension. Desorption into oil is indicated by a plus sign, and desorption into water is shown by a minus sign (Binks and Yin, 2016).

Several different types of interfacial stabilizers have been developed, including metallic oxide particles, metal-organic framework particles, anisotropic Janus particles (Mani et al., 2018) and segmented copolymers (Chen et al., 2015), (Röllig et al., 2019), (Ortiz et al., 2020), (Binks and Lumsdon, 2001). The most commonly used Pickering stabilizers are inorganic substances such as titania, silica, graphene, magnetic particles, and clay materials (Zhang and Wang, 2016), (Anupam, 2017), (French et al., 2015), (French et al., 2016), (Bashir et al., 2016). Cell encapsulation through a Pickering emulsion has numerous applications including in biocatalysis (Chen et al., 2015), (Röllig et al., 2019), environmentally-friendly bioremediation technology (Li et al., 2017), shielding from changes in pH (van Wijk et al., 2014), stirring-free biphasic enzymatic reactions (Wei et al., 2016), (Zhang et al., 2016), drug release (Zhang and Wang, 2016), 3D organ bioprinting applications for tumor models (Anupam, 2017), encapsulation of microbial cells to protect from pH changes (van Wijk et al., 2014), biopesticide formulations for *Bacillus thuringiensis* (Bashir et al., 2016) and for *Metarhizium brunneum* conidia (Yaakov et al., 2018), enzymatic reactions (Wei et al., 2016), (Zhang et al., 2016), cellular capsules based on liquid marbles (Zhang and Wang, 2016) and tissue engineering (Anupam, 2017). Most cell encapsulation technologies are passive, where the cell encapsulation process occurs randomly. In this case, the ability to encapsulate the cells is governed by their distribution in the encapsulation medium (Collins et al., 2015).

In our previous work, we developed a new strategy for single-cell encapsulation of an entomopathogenic fungus *M. brunneum* Mb7 that used an eco-friendly paraffin oil/water Pickering emulsion stabilized by silica NPs. This process led to a high distribution of these cells on plant foliage which also showed significantly greater *Spodoptera littoralis* larvae pest control than the control systems (Yaakov et al., 2018). However, the encapsulation yield was not sufficient.

The current study presents a new method for the determination of individual encapsulation yields *that determines the ratio of the number of cells that are individually encapsulated in the emulsion droplets out of the total number of cells in the emulsion.* Different mineral oil/water ratios and a range of concentrations of titania NPs functionalized by (3-aminopropyl)-triethoxysilane (APTES) Pickering emulsions were prepared. Titania dioxide (TiO_2_ or titania) NPs are eco-friendly Pickering stabilizers (Waghmode et al., 2019), with diameters of less than 100 nm (Negi et al., 2021). For example, ultrafine TiO_2_ is used in sunscreens because it can block UV radiation but stay transparent on the skin (Negi et al., 2021).

The stability of the emulsions was investigated by confocal microscopy and centrifugal analysis using a LUMiSizer^®^. A multisampling analytical centrifuge simultaneously measures the intensity of the transmitted light as a function of time and position over the whole sample length (Detloff et al., 2006). The most stable emulsions were then further studied for their individual encapsulation capability of *M. brunneum* Mb7 conidia. The obtained encapsulation yield, which was analyzed by confocal microscopy was as high as 44%.

## Materials and Methods

### Materials

Titania (AEROXIDE TiO_2_ P25, with an estimated primary particle size of 21 nm and a unique combination of anatase and rutile crystal structure was obtained from Evonik, Germany). The other components were obtained as indicated: (3-Aminopropyl) triethoxysilane (99% Sigma-Aldrich, United States), mineral oil (RTM-10, Sigma-Aldrich, United States), ultrapure deionized water (ULS/MS grade), Triton X-100 (nonionic surfactant, laboratory grade, Sigma-Aldrich, United States), ethanol absolute and Ethanol-96% (Bio Lab, Israel).

### Silanization of Titania Nanoparticles With APTES

Titania (5.63 g) was added to 130 ml of ethanol and stirred until there was complete dispersion. Next, 11 ml of APTES was added slowly to the solution. The reaction took place at ambient temperature for a duration of 1.5 h. Subsequent to silanization, the particles were centrifuged for collection (9,000 rpm, 10 min) and rinsed in ethanol four times. The titania-NH_2_ NPs were then vacuum dried at 35°C for 3 h.

### Preparation of the Titania-NH_2_ Pickering Emulsion

Pickering emulsions were derived from amine-functionalized titania in water and mineral oil. The titania-NH_2_ NPs were sonically dispersed in deionized water for 5 min (Sonics Vibra-Cell 750 W, 25% amplitude) at increasing titania-NH_2_ contents of 0.4, 1 and 2wt%. Next, mineral oil was added at oil/water ratios of 10:90, 30:70, 40:60 and 50:50 vol%, respectively. The mixture was subjected to sonication for 10 min to achieve emulsification (Sonics Vibra-Cell 750 W, 35% amplitude) and measurements were made to determine the average droplet diameter for each sample by implementing the Fiji software particle analysis tool applied to confocal microscopy images (Schindelin et al., 2012). Fifty droplets were sampled from each image and plotted as a 2D graph. The data were subjected to a one-way analysis of variance (ANOVA) and a Tukey-Kramer Multiple Comparisons Test using JMP (JMP Statistical Analysis Software, 2011; version 14, Macintosh, United States) for purposes of comparison at a *p* < 0.05 significance value.

### Instability Analysis

The instability index of creaming separation was analyzed using LUMiSizer^®^ software (L.U.M. GmbH, Berlin Germany), and calculated with the included software (SepView 6.0; LUM). The polycarbonate cuvettes with a 2 mm optical path length were filled with 400 µl of 40:60 and 50:50 vol% with 1wt% of titania-NH_2_ emulsions and were centrifuged in triplicate at 25°C simultaneously at a centrifugal force of 600 rpm (33 g). The transmission profiles were captured at 865 nm throughout the cell for 6 h (200 profiles every 5 s, 100 profiles every 10s, 100 profiles every 30 s and 600 profiles every 60 s).

### Sizes and Distributions of the Emulsion Droplets

The sizes and distributions of the oil droplets and titania-NH_2_ NPs were assessed using LUMiSizer^®^ software. The polycarbonate cuvettes with 2 mm optical path length were filled with 400 µl of 40:60 and 50:50 vol% with 1wt% of titania-NH_2_ diluted emulsions and were centrifuged at 25°C simultaneously at a centrifugal force of 300–4,000 rpm (8–1467 g). The transmission profiles were captured at 865 nm throughout the cell for 1 h (3 profiles were captured every 5 s from at 300–4,000 rpm (8–1467 g), 100 profiles every 30 s, 100 profiles every 120 s, 100 profiles every 280 s and 50 profiles every 360 s, at 4,000 rpm (1467 g). The particle size analysis (PSA) was run according to the density of the mineral oil and titania-NH_2_ NPs using LUMiSizer^®^ software.

### Fungal Material and Preparation

*Metarhizium brunneum* Mb7-GFP (Mb7) (Ment et al., 2012) mutants were cultured on Sabouraud dextrose agar (SDA) (Difco, Becton−Dickinson, MD) for 2 weeks at 28°C until sporulation was achieved. The fungal colony was scraped to harvest the conidia to make conidial suspensions. Then the collected material was suspended in sterile distilled water with 0.01% Triton X-100 followed by vortexing. After filtering the suspension through three layers of gauze, a hemocytometer was used to assess conidial concentrations.

### Encapsulation of Conidia in the Titania-NH_2_ Pickering Emulsion

Two different oil/water ratios were selected for the single-cell encapsulation in a Pickering emulsion for a 1wt% titania-NH_2_ NPs content: 40:60 and 50:50. The 390, 400, 000 Mb7-GFP conidia were suspended in 300 µl of 0.02% Triton X-100, followed by 5 min vortexing. Afterward, 45 µl of the suspension was added to 1.5 ml of the emulsions. After the mixture was vortexed at high speed in vortex mixers for 1 s, samples (2 μl) were placed on glass slides and examined by confocal microscopy.

### Efficiency Calculation of Conidia Encapsulation in Titania-NH_2_ Pickering Emulsion

To calculate the yield of conidia encapsulation in the Pickering emulsion, 10 confocal images were captured from each slide (for a total number of slides of 3–5). To calculate the efficiency of conidium encapsulation in the 1 µl of emulsion, the initial number of conidia (before encapsulation) was calculated and the number of encapsulated conidia was counted in each image. To calculate the efficiency of the conidium encapsulation the ratio of the concentration of conidia in 1 µl after encapsulation/concentration of conidia in 1 µl was calculated before encapsulation X 100%.

### Procedure for Confocal Laser Scanning Microscopy and Image Analysis

The samples were subjected to laser scanning confocal microscopy. The fluorescence emission of GFP was recorded at 500–525 nm. A Leica SP8 laser scanning microscope (Leica, Wetzlar, Germany) equipped with a solid state laser with 488 nm light, HC PL APO CS 20×/0.75 objective (Leica, Wetzlar, Germany) and Leica Application Suite X software (LAS X, Leica, Wetzlar, Germany) were used for 3D image acquision. The GFP signal was imaged using an argon laser. The emission was detected in a 500–525 nm range. To tabulate the *Metarhizium* conidia, image stacks were displayed as a Z projection (at maximum intensity) to locate all the fluorescent conidia, and then were counted by a Fiji software cell counter.

### Statistical Analysis

All statistical analyses were calculated with the JMP package (SAS Institute, 2011). A one-way ANOVA followed by a Tukey-Kramer honestly significant difference (HSD) tested for comparisons between all pairs for the number of conidia on the slides.

## Results and Discussion

### Preparation of Titania-NH_2_ Pickering Emulsion

Titania NPs were functionalized by APTES by silanization to introduce amine groups on the surface of the NPs (Pasternack et al., 2008). The silanization procedure which we have used is well established in the literature, as the resulting particle have already show their ability to serve as Pickering stabilizers for oil-in-water emulsions in our previous works (Feldbaum et al., 2021). Oil-in-water Pickering emulsions stabilized by amine-functionalized titania NPs were prepared. Different amine functionalized titania content and oil/water ratios were implemented to determine the optimal conditions for a stable Pickering emulsion system that would meet the requirements of single-cell encapsulation. The titania-NH_2_ content varied (0.4, 1, and 2wt%) at oil/water ratios of 10:90, 30:70, 40:60, and 50:50 respectively.

**FIGURE 1:**
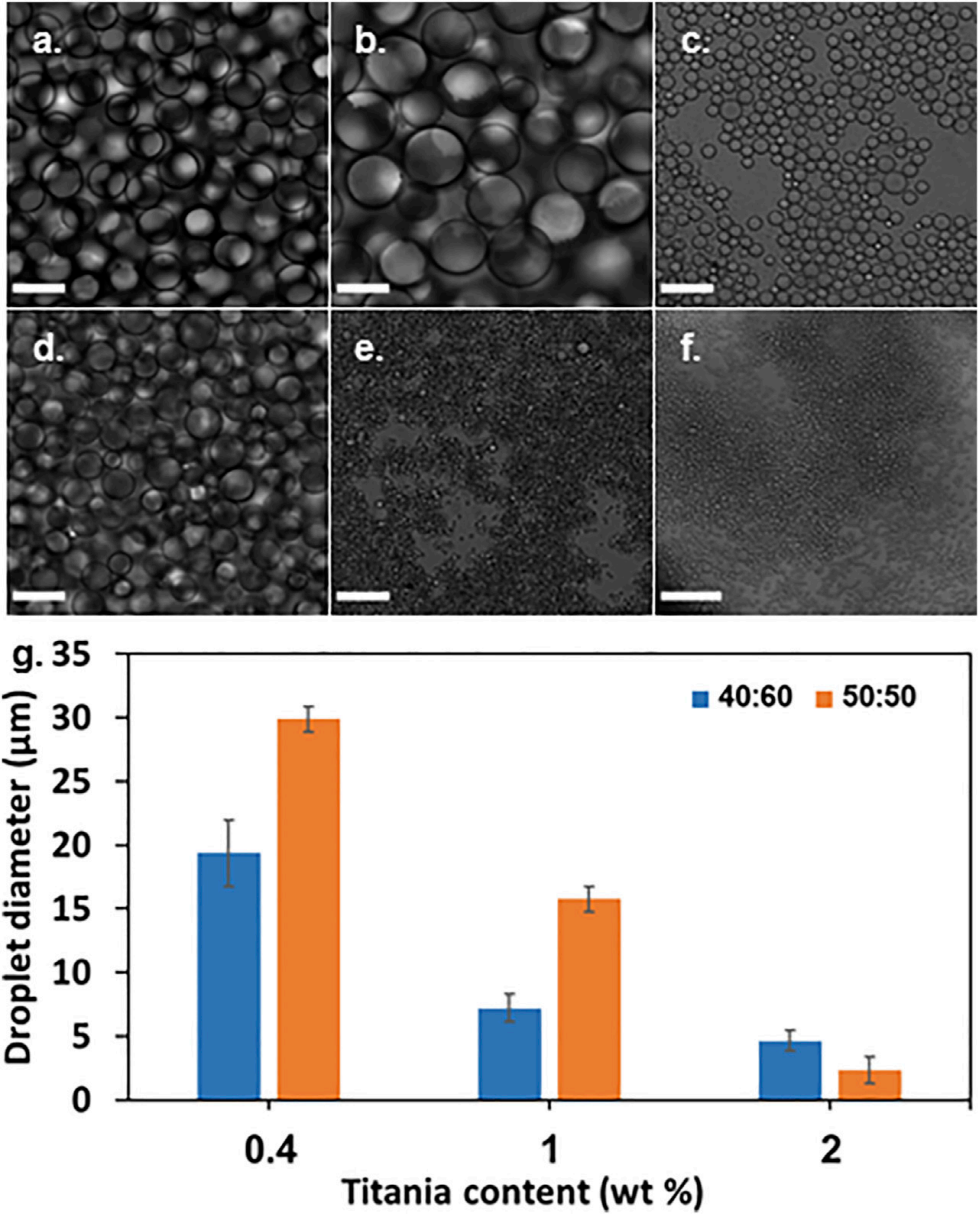
Pickering emulsions with 0.4, 1, and 2wt% titania-NH_2_ content at different oil/water ratios. **(A)** 40:60, 0.4wt%. **(B)** 50:50, 0.4wt%. **(C)** 40:60, 1wt%. **(D)** 50:50, 1wt%. **(E)** 40:60, 2wt%. **(F)** 50:50, 2wt%. **(G)** Droplet diameter as a function of titania-NH_2_ content (wt%) and oil percentages in the emulsion (vol%). Scale bar is 30 µm.

**FIGURE 2:**
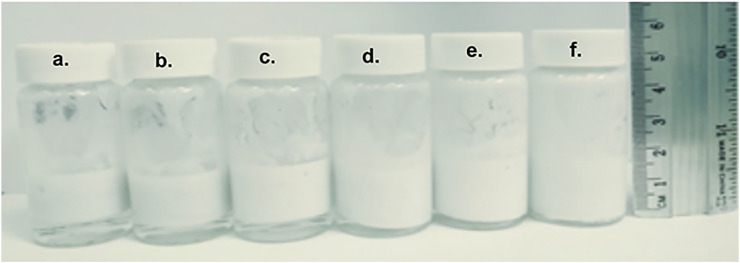
Characterization of the titania-NH_2_ Pickering emulsion oil/water ratio with NPs content of wt%: **(A)** 40:60, 0.4wt%. **(B)** 50:50, 0.4wt%. **(C)** 40:60, 1wt%. **(D)** 50:50, 1wt%. **(E)** 40:60, 2wt%. **(F)** 50:50, 2wt%.

Confocal microscopy micrographs of emulsions with 0.4, 1, and 2wt% titania-NH_2_ NPs at different oil/water ratios are depicted in Figure 1. The diameter of the mineral oil droplets were highly tunable and could be varied widely in a range of 2–30 µm. Figure 2G depicts the average mineral oil droplet diameters (weighted average) of the emulsions versus the content of the titania-NH_2_ NPs at two different mineral oil/water ratios. The analysis showed that the higher the titania-NH_2_ content, the smaller the droplet diameter of the mineral oil, due to the increase of the total surface area of the oil/water interface (Frelichowska et al., 2010). In addition, under a given content of titania-NH_2_, the increase of the volume fraction of the mineral oil resulted in larger droplet sizes (Frelichowska et al., 2010). Thus, this system exhibited normal behavior in terms of the relationship between the droplet sizes and the oil/water volume ratios (Yaakov et al., 2018), (Linke and Drusch, 2018), (Frelichowska et al., 2010), (Qi et al., 2014), (Cohen et al., 2019). Adjusting the emulsion composition also served to fine-tune the resulting droplet size, which is essential for individual conidium encapsulation. The relatively high stability of the Pickering emulsion can thus be ascribed to the low coalescence rate of the droplets.

A LUMiSizer^®^ (LUM GmbH, Germany) assessed the stability of the Pickering emulsions over time, confirming visual inspection. Most of the prepared emulsions were stable for 7 days marked by the check √ (see Table 1). A titania-NH_2_ content of 0.4, 1 and 2wt% at oil/water ratios of 40:60 and 50:50 oil/water (vol%) had the highest stability, which were maintained for more than 5 months (Figure 2).

**TABLE 1:** Composition of the emulsions.

oil:water/TiO_2_-NH_2_ [wt%]	10:90	30:70	40:60	50:50
0.4	-	-	√	√
1	-	-	√	√
2	-	-	√	√

**FIGURE 3:**
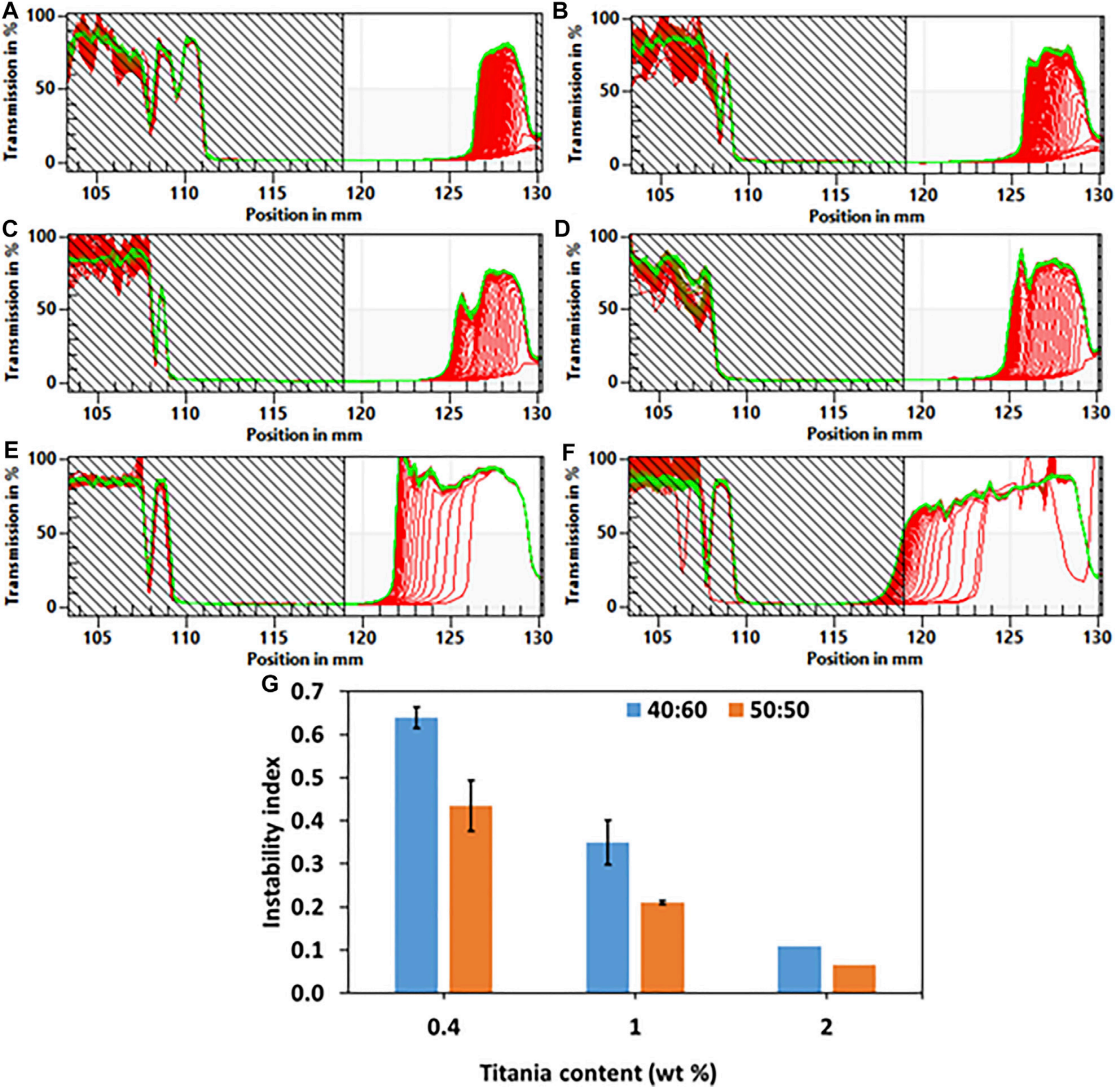
Light transmission curves of Pickering emulsions with 2, 1, and 0.4wt% titania-NH_2_ at different oil/water ratios. **(A)** 40:60, 2wt%. **(B)** 50:50, 2wt%. **(C)** 40:60, 1wt%. **(D)** 50:50, 1wt%. **(E)** 40:60, 0.4wt%. **(F)** 50:50, 0.4wt%. **(G)** The instability index of Pickering emulsions as a function of titania-NH_2_ content (wt%) and oil percentages in the emulsion (vol%). In 2wt% titania-NH_2_ based emulsion have lower standard deviation. Green lines represent the latest measured intensity profiles while red lines represent initial profiles.

The stability of the emulsions was characterized quantitatively by calculating the instability index using a LUMiSizer^®^ (LUM GmbH, Germany). Light transmission was measured during centrifugation of the emulsions, which were added to cuvettes at 25°C. The transmission profiles were used to characterize the stability of the emulsions. Figures 3A–F depicts the transmission profiles; i.e., the percentage of transmitted light versus the position in the cuvette. Creaming was identified in all the emulsions. The results showed that the higher the titania-NH_2_ content in the emulsion, the lower the instability index for all compositions (Figure 3G). Emulsions with 2wt% titania-NH_2_ content at different oil/water ratios were found to be the most stable (Ruiz-Rodriguez et al., 2014). This is due to the increase in the total surface area of the oil/water interface that creates a distinct steric barrier that limits coalescence, flocculation and Ostwald ripening (Ruiz-Rodriguez et al., 2014). The instability index of the 50:50 and 40:60 oil/water ratio emulsions was not significantly different for any of the titania-NH_2_ contents. Bancroft’s rule (Ortiz et al., 2020) states that emulsions with a greater likelihood for high stability tend to be formed when mixing the same volume of oil and water. In the current study, the stable emulsion fraction was increased progressively starting with oil/water ratios exceeding 0.5, and was decreased with oil/water ratios below 0.50 (Ortiz et al., 2020). Thus, the emulsions behaved according to Bancroft’s rule (Ortiz et al., 2020).

The most stable emulsion was obtained with 2wt% titania-NH_2_ at an oil/water ratio (vol%) of 50:50 which had the smallest droplet size and the lowest instability index. This can also be ascribed to the increase in the total surface area of the oil/water interface (Yaakov et al., 2018), (Zhang et al., 2016), (Zhang and Wang, 2016), (Anupam, 2017), (Weiss et al., 2020), (Ye et al., 2017), (Schröder et al., 2018), the wettability of particles and the dispersed-phase volume (Xiao et al., 2016).

However, this emulsion is not suitable for individual encapsulation of conidium because the size of the droplets is too small (∼2 µm) to host the conidium which are ∼4 µm in length. Therefore, emulsions with 1wt% titania-NH_2_ at 40:60 and 50:50 oil/water (vol%) ratios were selected for the encapsulation experiments given their high stability and suitable droplet size.

**FIGURE 4:**
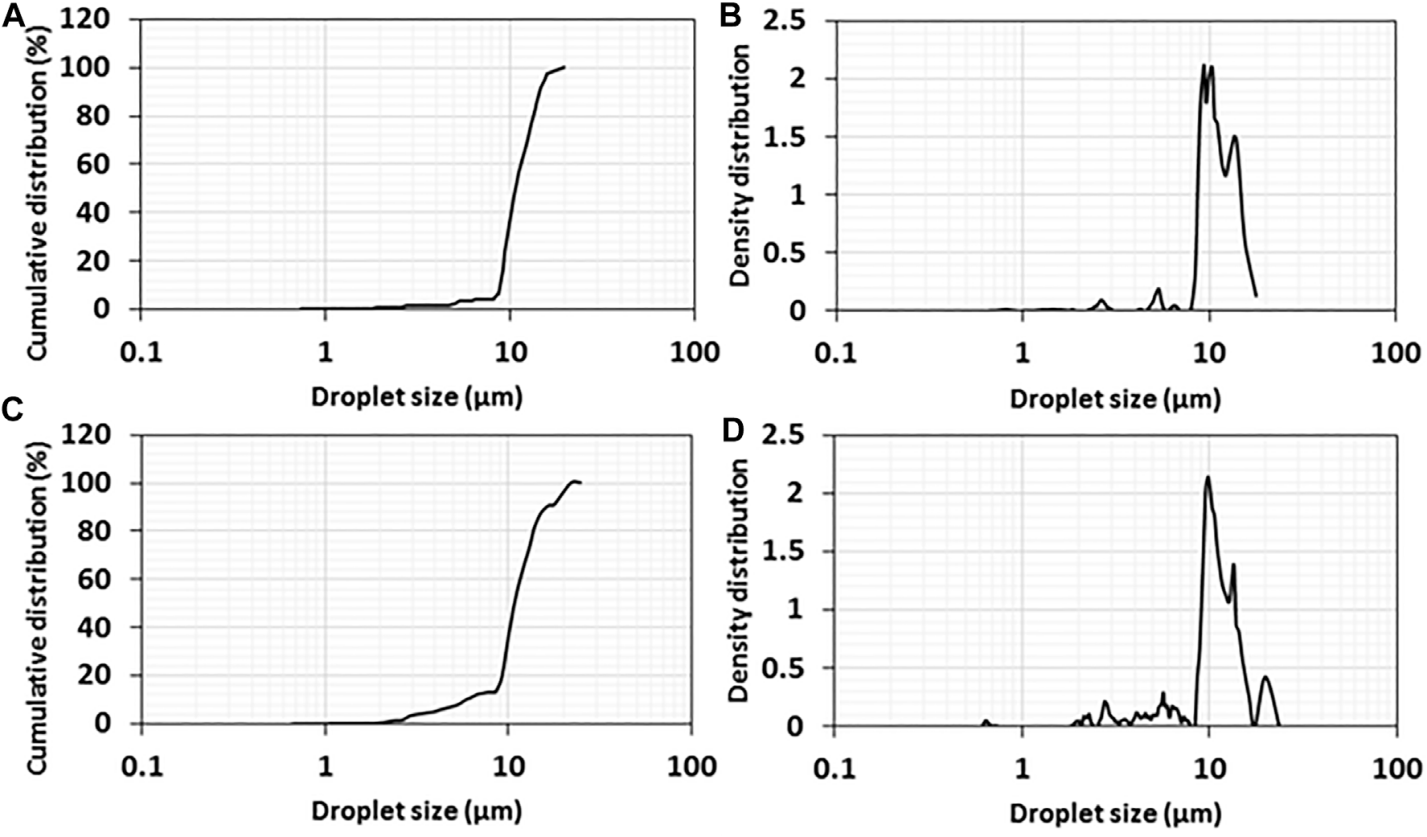
Cumulative and density distributions of oil droplet sizes in Pickering emulsions with a 1wt% titania-NH_2_ NPs content at **(A, B)** 40:60 and **(C, D)** 50:50 oil/water (vol%) ratios.

The droplet size of the emulsions of 1wt% titania-NH_2_ content at 40:60 and 50:50 oil/water (vol%) ratios was characterized by the LUMiSizer^®^. Figure 4 presents the mean cumulative distribution values for the three positions at 109 mm, 111 mm, and 113 mm (“constant position”) in the cuvette. It shows that 90% of the droplets of the emulsions at a 40:60 oil/water ratio (vol%) were smaller than 14 µm (Figures 4A,B). The average droplet size for this emulsion was 11 ± 2 µm in the confocal analysis (Figure 2G). It can be seen that 90% of the droplets of emulsions with an oil/water ratio (vol%) of 50:50 measured less than 17 µm (Figures 4C,D). The average droplet size for this emulsion was 10 ± 4 µm in the confocal analysis (Figure 2G). The size of the droplets in the emulsion at a 40:60 oil/water ratio was smaller than at a 50:50 oil/water ratio (the graph of the emulsion at 50:50 oil/water ratio is broader than the emulsion at a 40:60 oil/water ratio). Hence, these results are consistent with the droplet size analysis (Figure 2). Both Figures 2, 4 shows that the studied emulsions have relativity low polydispersity of the droplet size at any studied composition, which makes them suitable for conidia individual encapsulation because that the property of individual encapsulation is governed by the droplet size of the emulsions (Yaakov et al., 2018), (Feldbaum et al., 2021).

### Single-Cell Encapsulation

**FIGURE 5:**
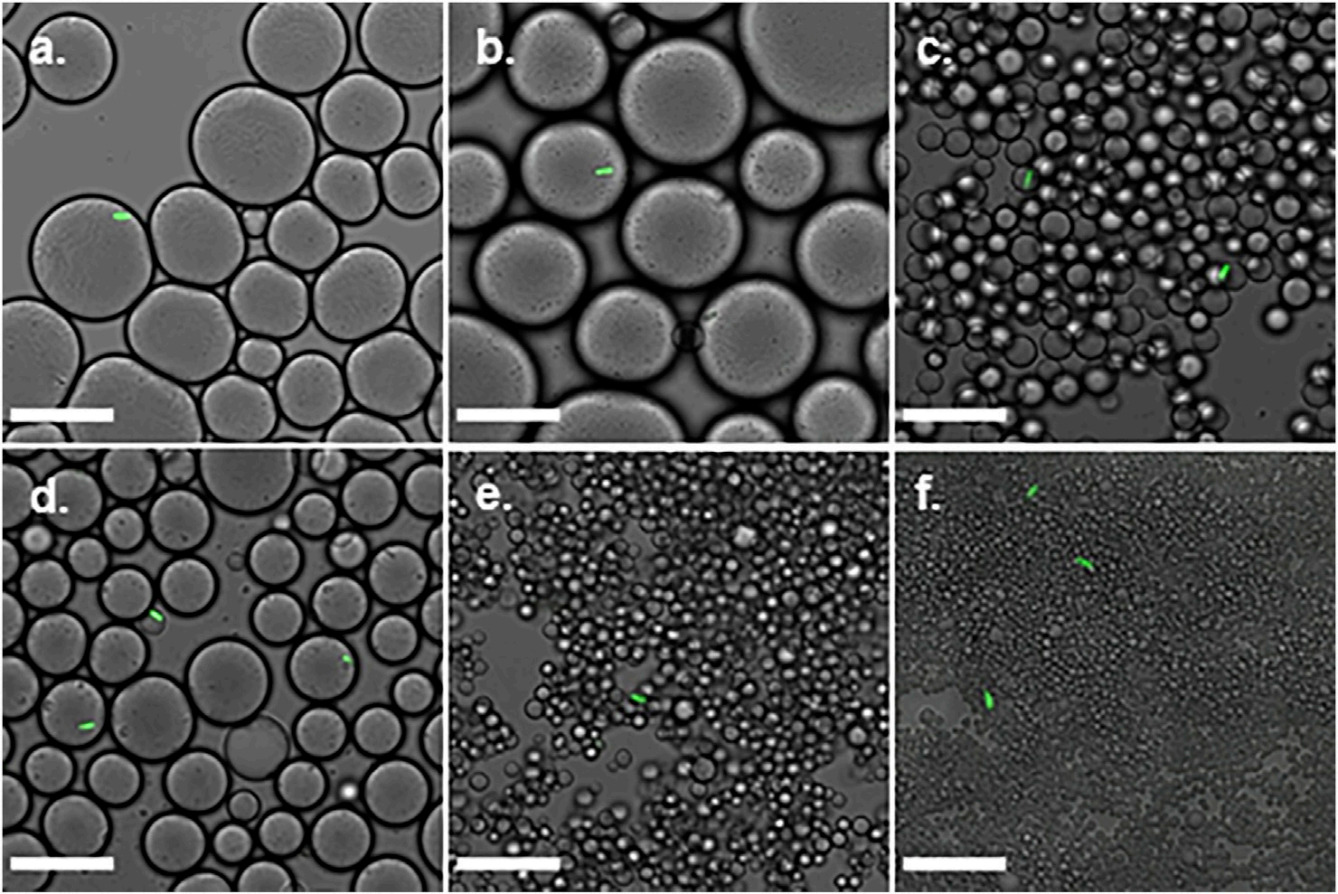
Confocal microscopy images of single-cell encapsulation of 0.02% Triton X-100 *Metarhizium brunneum* Mb7-GFP conidia suspension in titania-NH_2_ Pickering emulsion oil/water ratio. **(A)** 40:60, 0.4wt%. **(B)** 50:50, 0.4wt%. **(C)** 40:60, 1wt%. **(D)** 50:50, 1wt%. **(E)** 40:60, 2wt%. **(F)** 50:50, 2wt%. Scale bar is 30 μm.

Mb7-GFP conidia were added to the titania-NH_2_ Pickering emulsions. Confocal microscopy (Figure 5) confirmed the individual encapsulation of conidia in the mineral oil phase of the emulsions. Single-cells were successfully encapsulated in the mineral oil droplets in emulsions with 1wt% NPs at ratios of 40:60 and 50:50 oil/water (vol%) (Figures 5C,D). In these emulsions, the droplet concentration was approximately one order of magnitude greater than the conidia concentration, since 3–21 conidia droplets were seen in the confocal microscopy images even at very low magnification. Figure 5 illustrates some characteristic confocal microscopy images that clearly confirm the single-cell conidium encapsulation in the mineral oil droplets of the emulsions. The emulsions that demonstrated single-cell encapsulation (at titania-NH_2_ contents of 1wt%, at 40:60 and 50:50 oil/water (vol%) ratios) had a mean droplet diameter of 7 ± 1, and 16 ± 3 μm, respectively (Figure 2E), which approximates the size of the conidia, which are ∼4 μm in length. These results shows that individual encapsulation is obtained when the droplet sizes and the cell sizes are of the same order of magnitude. As was also shown in our previous works (Yaakov et al., 2018), (Feldbaum et al., 2021). The conidia assemble at the oil phase in the water/oil biphasic system as a result of their hydrophobic surface (Boucias and Pendland, 1998), (Luke et al., 2015).

The titania-NH_2_ Pickering emulsions were prepared by an ultrasonic probe. Exposing a liquid sample to ultrasonic waves produces vigorous agitation characterized by high shear forces that cause emulsification. However, ultrasonication cannot be achieved in the presence of conidia because it leads to cell lysis. For this reason, the conidia were only incorporated after the emulsion had been gently agitated by vortexing, which enabled the conidia to penetrate the mineral oil droplets. French et al. discussed the way in which the application of shear forces to the system enables cells or particles to enter the droplets of Pickering emulsions. (French et al., 2015; French et al., 2016). Specifically, particles in the oil/water interface undergo transformations between the droplets when shear forces are applied, which leaves temporary voids at the interface. This may explain why gentle agitation, which likely produced sufficient shear forces, led to the successful encapsulation of the conidia in the mineral oil droplets. Using NPs that were relatively small in diameter (∼22 nm) for the preparation of the Pickering emulsions may also have lessened their ability to create a barrier at the interface impeding penetration of the conidia, since they are two orders of magnitude larger (∼4 μm in length) than the NPs.

### Encapsulation Yield

Although that there are many methods which enables to quantify the number of conidia in the emulsions, such as measurement of DNA or RNA by UV-vis, However, methods which allow to quantify how many cells are individually encapsulated in the oil droplets of an emulsion are currently out of reach. To this end, in the current study, we have developed a method, which enables to directly measure and quantify the yield of the cells, which are individually encapsulated in the oil droplets of the emulsion. The method is based on direct counting of the individually encapsulated cells via confocal microscopy. Our method would enable to characterize the encapsulation yield of cells in almost any type of emulsions. Thus, a major uniqueness of the current work is the development of a simple and reliable method to quantify the individual encapsulation yield of cells in emulsions.

The encapsulation yield was defined as the percentage of conidia that were individually encapsulated out of the total number of cells. The Mb7 conidia were suspended in a Triton X-100 surfactant to prevent aggregation due to the hydrophobic interactions between the cells (Jeffs and Khachatourians, 1997) that increases the dispersion of the conidia in the emulsion and the spread on the surface. The same volumes of the suspension were added to the emulsions and mixed by brief vortexing. Confocal microscopy micrographs of Pickering emulsions are depicted in Figure 5. It shows that the single-cell encapsulation performance depended on the droplet size. The most effective single-cell encapsulation was observed in emulsions with a 1wt% titania-NH_2_ content where each droplet covered a single conidium entirely. The encapsulation in emulsions with 2wt% NPs was unsuccessful because the small size of droplets was incompatible with the size of the conidia. Emulsions with 0.4wt% titania-NH_2_ contained one or more conidia per droplet (data not shown) since the droplets were larger than the conidia. Therefore, emulsions with 1wt% titania-NH_2_ content at 40:60 and 50:50 oil/water (vol%) ratios were chosen for the determination of the single-cell encapsulation yield.

The encapsulation yield was measured and characterized by confocal microscopy and analyzed by Fiji software. Each sample was studied based on five different slides of 1 ml emulsion with conidia, with 10 confocal fields for each slide. The concentration ( $C_{\mathrm{single}}$ ) of individually encapsulated conidium was directly counted in the confocal microscopy fields measuring 188 × 188 µm with a thickness of 10 ± 2 μm, and 15 ± 3 μm at 40:60 and 50:50 oil/water (vol%) ratios respectively (each rectangular field had a specific thickness). The concentration of individually encapsulated conidium ( $C_{\mathrm{single}}$ ) for each emulsion was calculated by dividing the average number of individually encapsulated conidium in each confocal microscopy field by the field volume. The single conidium encapsulation yield $Y_{\mathrm{single}}$ was defined as the concentration of encapsulated conidium ( $C_{\mathrm{single}}$ ), divided by the total concentration of conidia $C_{0}$ , in the emulsion, as can be seen in Eq. (1). Detailed calculations of the total concentration of conidia in 1 ml of emulsion appear in the supporting information.

$$Y_{\mathrm{single}=\;\frac{C_{\mathrm{single}}}{C_{0}}}$$ (1)

**FIGURE 6:**
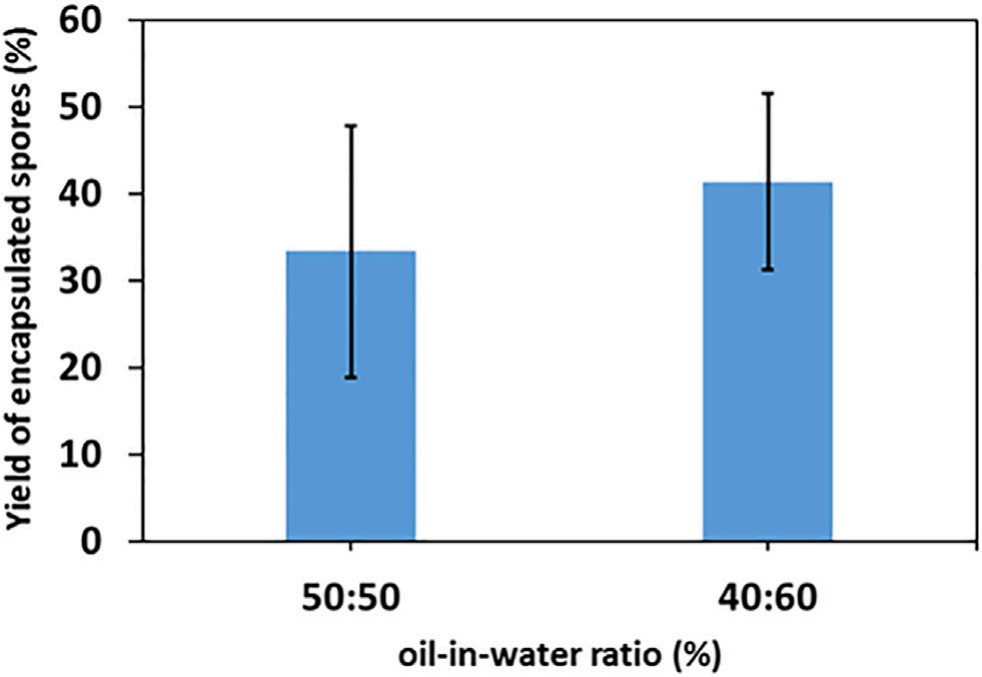
Encapsulation yield of single-cell encapsulation of 0.02% Triton X-100 *Metarhizium brunneum* Mb7-GFP conidia suspension with a 1wt% titania-NH_2_ content Pickering emulsion at oil/water 40:60 and 50:50 (vol%) ratios.

The yields of single-conidium encapsulation in the two main emulsions with oil/water ratios of 40:60 and 50:50 oil and 1wt% of titania-NH_2_ are depicted in Figure 6. The yields were 33 ± 14% at a 40:60 emulsion and 41 ± 10% at a 50:50 emulsion. The encapsulation rate increased at higher oil ratios in the emulsion. The encapsulation mechanism of the conidia in the o/w Pickering emulsion remains unclear and will be investigated in future research. There were only a few cases of double conidia encapsulation or triple conidia encapsulation that were detected in our yield characterization assay, which also reflects the efficiency of our method for the individual encapsulation of conidia.

The structure property relationship of the studied emulsions was comprehensively investigated in terms of droplet size vs composition and stability (as can be seen in Figure 2 and Figure 3 respectively), to meet the demands of high yield individual encapsulation of the conidia cells as can be seen in Figure 6. First screening of the emulsion compositions have shown that six different compositions have shown the highest stability. We have found in our previous works that the key parameter for successful conidium encapsulation. The average droplet size of the six stable emulsions have been characterized. It was found that at 1wt% titania-NH_2_ and o/w ratios of 40:60 and 50:50 had an average droplet diameter of 7 ± 1, and 16 ± 3 μm, respectively, which were closest to the size of the *Metarhizium Brunneum* conidia, which are approximately 4 μm in length.

The role of the encapsulation of conidia in the studied Pickering emulsions is passive in its nature, and is governed by thermodynamics, indeed the assembly of the conidia cells in the oil droplets is affected and enhanced by the hydrophobic nature of their surface (Yaakov et al., 2018), (French et al., 2015), (French et al., 2016). However, introduction of shear force to the system is do required in order to enhance the ability of cells or particles to enter into the droplets of Pickering emulsion and to overcome the kinetic barrier. Indeed, according to (French et al., 2016), (French et al., 2015), the shear forces which are developed during emulsification, lead to changes in the positions of the particles at the oil/water interface, leaving temporary voids at the interface, which might enable the penetration of the cells into the droplets. Overall, the exact penetration mechanism of cells into the droplet of Pickering emulsion is still under investigation.

## Conclusion

This work describes a highly effective innovative method for the individual encapsulation of fungal conidia in the droplets of a titania-NH_2_ based mineral oil-in-water Pickering emulsion at significantly high individual encapsulation yield. The emulsions remained stable for over 5 months. The structure and stability of the emulsions in terms of their composition were comprehensively investigated by confocal microscopy and the LUMiSizer^®^. The most stable compositions with a droplet size suitable for single-conidium encapsulation were further studied for their individual encapsulation capability. *Metarhizium brunneum* conidia an extensively investigated entomopathogenic fungus and biopesticide, were mixed into the Pickering emulsions. We have developed in this study a method to characterize the individual encapsulation of cells in emulsions via confocal microscopy. Our encapsulation approach enables the compartmentalization of the cells individually at a relatively high yields reaching to 44%. This method thus constitutes a rapid and easy to use approach for individual encapsulation of conidia and cells in general at significantly high yields by eco-friendly titania, based on commonly used industrial emulsification and agitation techniques.

## Data Availability

The original contributions presented in the study are included in the article/supplementary material, further inquiries can be directed to the corresponding author.
